# A Deformation-Based Shape Study of the Corpus Callosum in First Episode Schizophrenia

**DOI:** 10.3389/fpsyt.2021.621515

**Published:** 2021-06-04

**Authors:** Weikai Huang, Minhua Chen, Guiwen Lyu, Xiaoying Tang

**Affiliations:** ^1^Department of Electrical and Electronic Engineering, Southern University of Science and Technology, Shenzhen, China; ^2^Department of Radiology, The First Affiliated Hospital of Shenzhen University, Shenzhen, China

**Keywords:** first-episode schizophrenia, gender effect, corpus callosum, shape analysis, deformation, sub-region

## Abstract

**Background:** Previous first-episode schizophrenia (FES) studies have reported abnormalities in the volume and mid-sagittal size of the corpus callosum (CC), but findings have been inconsistent. Besides, the CC shape has rarely been analyzed in FES. Therefore, in this study, we investigated FES-related CC shape abnormalities using 198 participants [92 FES patients and 106 healthy controls (HCs)].

**Methods:** We conducted statistical shape analysis of the mid-sagittal CC curve in a large deformation diffeomorphic metric mapping framework. The CC was divided into the genu, body, and splenium (gCC, bCC, and sCC) to target the key CC sub-regions affected by the FES pathology. Gender effects have been investigated.

**Results:** There were significant area differences between FES and HC in the entire CC and gCC but not in bCC nor sCC. In terms of the localized shape morphometrics, significant region-specific shape inward-deformations were detected in the superior portion of gCC and the anterosuperior portion of bCC in FES. These global area and local shape morphometric abnormalities were restricted to female FES but not male FES.

**Conclusions:** gCC was significantly affected in the neuropathology of FES and this finding was specific to female FES. This study suggests that gCC may be a key sub-region that is vulnerable to the neuropathology of FES, specifically in female patients. The morphometrics of gCC may serve as novel and efficient biomarkers for screening female FES patients.

## Introduction

Schizophrenia is a chronic and severe mental disorder that in general presents with paranoid delusions and auditory hallucinations (1). The social disability and cost induced by schizophrenia are very large (2, 3). In 2013, schizophrenia is listed as one of the top 25 causes of global disability (4). The World Health Organization estimated that in Western countries the direct costs of schizophrenia account for 1.6–2.6% of the total health care expenditures and the economic burden of schizophrenia exceeds $60 billion per year in the United States (5, 6).

There is evidence showing that the early stages of schizophrenia are essential for the formation and prediction of disease course and outcome (7). First-episode schizophrenia (FES) is the stage at which individual first presents symptoms that are consistent with those of schizophrenia and formally receives a clear diagnosis of schizophrenia after professional evaluation. Consequently, FES has been the focus of clinical research which typically occurs in the late teenage years or the early 20s (8). Brain's structural abnormalities have been identified in FES. A large number of neuroimaging studies on structural brain abnormalities in FES focused on the gray matter, including the hippocampus, the entorhinal cortex, and the insular cortex (9–15). For white matter, most FES studies analyzed a range of white matter tracts instead of a single one (16–18). The research on corpus callosum (CC) in FES is comparatively rare, especially morphological analyses of CC (19–23).

The CC is the largest white matter structure in the human brain and the largest commissural fiber bundle connecting the two cerebral hemispheres (24). Schizophrenia-associated morphological abnormalities of CC, especially those of its mid-sagittal section, have been reported in various magnetic resonance imaging (MRI) studies (25–28). However, the findings on FES are inconsistent. A number of FES studies have reported area reductions in the 2D mid-sagittal section of CC and its subdivisions, when compared to healthy controls. For example, Collinson et al. (29) discovered that compared with a control group both area of the 2D mid-sagittal section and volume of the entire 3D CC decreased significantly in FES but there were no significant gender difference in either the patient group or the control group. Bachmann et al. (19) found smaller areas in CC and CC's subdivisions in FES compared to controls and the CC area in women was larger than that in men within both the patient group and the control group. Keshavan et al. (20) observed smaller areas in CC, particularly in the isthmus and anterior parts of genu, body and splenium, in FES compared to a control group and there was no age-related increase in the CC area in FES which nevertheless had been observed in the control group. Some studies discovered that both the 3D CC volume and the 2D CC area did not differ significantly between FES patients and healthy controls (30). Also, the integrity of CC was not disrupted in FES (21). A plausible conjecture for those inconsistent results is that the CC morphology is tightly linked to the duration of illness in FES and there may be no reduction in the volume or area of CC in FES patients with a duration of illness <1 year (31).

Most of the aforementioned researches focused on the 2D mid-sagittal section of CC, but not the overall shape. In recent years some shape measures have been proposed. For example, Walterfang et al. (32) observed that FES had subtle thickness reductions in the genu of CC. However, the majority of existing CC shape measures ignored the potential deformation information around the neighborhood and only explored the 2D CC boundary features based on isolated points. To address this limitation, in this study, novel deformation-based shape analyses have been performed to analyze localized 2D CC morphometrics, in the framework of large deformation diffeomorphic metric mapping (LDDMM). Compared to area and thickness measures, the curve shape may better reflect the subtle morphometric abnormalities of CC.

LDDMM is one of the state-of-the-art anatomical manifold matching algorithms that had been successfully applied to characterize localized morphometrics of different structures of interest in various neurodegenerative disorder studies (33–36). LDDMM is a general registration framework that can match manifolds of different dimensions, such as landmarks, curves, surfaces, and dense images. In the task of analyzing the shape of a 2D object, such as the mid-sagittal section of CC, LDDMM for curves (LDDMM-curve) (37) is an appropriate choice. Indeed, in one of our previous studies (38), we have already successfully applied LDDMM-curve to investigate the region-specific morphological abnormalities of CC and its sub-regions in Alzheimer's disease.

In this study, the CC was divided into three sub-regions, the genu (anterior), body and splenium (posterior), namely gCC, bCC, and sCC, to identify the key sub-regions of CC targeted by the neuropathology of FES. We first examined the areas of the 2D CC section and its three sub-regions. After that, we extracted deformation markers (point-wise Jacobian determinants) from the diffeomorphic transformations from LDDMM-curve to serve as the localized shape characteristics of CC and then compared those localized shape characteristics between a FES group and a control group. The primary goal of this work is to analyze the shape of the mid-sagittal CC curve and to shed new insights to FES-associated CC abnormalities.

Several studies have identified gender differences in the psychopathological characteristics and social functioning of FES patients (30, 39, 40). A previous study has reported a specific gender differences in schizophrenia; namely, males seemed to exhibit more negative symptoms whereas females displayed more affective symptoms, auditory hallucinations, and persecutory delusions (41). In addition, some studies discovered that after correcting for the confounding effect of brain size females had larger mid-sagittal CC areas than males (42). Walterfang et al. (30) also reported that females had significantly larger CC areas than males in a control group and that female patients with psychosis had larger CC area reductions than male patients. These observations may suggest that there is significant gender effect in the neuropathology of FES. Based on findings from previous studies, we hypothesize that CC's morphology is more susceptible to the neuropathology of FES in female than in male. Specifically, we anticipate more significant morphological abnormalities of CC in female patients than in male patients when compared to their corresponding healthy control counterparts.

## Materials and Methods

### Participants

In this study, all FES patients were recruited from the First Affiliated Hospital of Shenzhen University between March 2008 and November 2018. Consensus diagnosis of FES was reached based on the Diagnostic and Statistical Manual of Mental Disorders, Fourth Edition (DSM-IV) diagnosis criterion (43). Exclusion criteria included history of substance dependence, other diseases of the central nervous system, or unstable medical conditions. In order to eliminate the confounding effect of neuroleptic medication, all FES patients were antipsychotic-naïve. Meanwhile, we recruited age and gender matched healthy controls (HCs). All participants volunteered to be part of this research and had the right to withdraw at any time. Informed consents were obtained from all participants or family relatives, as approved by the Ethics Committee at the First Affiliated Hospital of Shenzhen University.

The demographic information of all participants is shown in Table 1. A total of 198 participants were involved, including 92 FES patients (50 females and 42 males) and 106 HC participants (47 females and 59 males), aged from 12 to 43 years (FES patients' average age = 22.40 ± 5.59 years; HC participants' average age = 23.68 ± 4.04 years). There is no significant group difference on either sex (*P* = 0.16) or age (*P* = 0.071) or duration of education (*P* = 0.43). The Global Assessment of Function (GAF) (44) and the Positive and Negative Syndrome Scale (PANSS) (45) were used to, respectively, assess the severity of FES patients in terms of social function and clinical symptoms.

**Table 1:** Demographic information.

**Variables**	**FES patients**			**Healthy controls**		
	**All (*n* = 92)**	**Male (*n* = 42)**	**Female (*n* = 50)**	**All (*n* = 106)**	**Male (*n* = 59)**	**Female (*n* = 47)**
Age (years)	22.40 ± 5.59 (12, 43)	23.48 ± 6.01 (14, 43)	21.50 ± 5.10 (12, 36)	23.68 ± 4.04 (18, 43)	24.14 ± 4.77 (18, 43)	23.11 ± 2.84 (18, 29)
Duration of education (years)	12.32 ± 3.18 (6, 20)	12.74 ± 3.35 (6, 20)	11.95 ± 3.02 (7, 18)	13.85 ± 2.81 (8.5, 20)	13.70 ± 3.01 (8.5, 20)	14.07 ± 2.52 (10, 18)
Age of onset (years)	21.26 ± 5.29 (12.8, 42.7)	22.52 ± 5.83 (14.9, 42.7)	20.09 ± 4.51 (12.8, 34.7)			
Duration of untreated psychosis (months)	14.66 ± 23.43 (3, 147)	13.01 ± 24.97 (3, 147)	16.17 ± 22.16 (3, 123)			
GAF scores	28.46 ± 9.40 (5, 65)	26.62 ± 7.64 (5, 43)	30.26 ± 10.66 (16, 65)			
**PANSS scores:**						
Total	93.90 ± 16.24 (61, 149)	93.26 ± 16.39 (71, 149)	94.49 ± 16.30 (61, 127)			
Positive	24.55 ± 6.31 (7, 38)	25.88 ± 5.74 (17, 38)	23.32 ± 6.63 (7, 37)			
Negative	20.89 ± 8.46 (7, 49)	18.53 ± 8.34 (7, 49)	23.05 ± 8.07 (10, 37)			
General psychopathology	48.46 ± 8.39 (31, 67)	48.85 ± 7.63 (36, 63)	48.10 ± 9.12 (31, 67)			

### MRI Data Acquisition

All structural MRI data were acquired from a Siemens Trio Tim 3T scanner. For each participant, its whole-brain T1-weighted 3D volume image was obtained using a magnetization prepared-rapid acquisition gradient echo (MPRAGE) sequence with the following scanning parameters: repetition time = 13.40 ms, echo time = 4.6 ms, flip angle = 20, field of view = 256 × 256, and 1-mm^3^ isotropic resolution across the entire cranium. All MR images were visually examined by one neuroradiologist for data quality control.

### CC Segmentation

In this study, for each T1-weighted brain image, a validated fully-automatic segmentation algorithm, namely the multi-atlas likelihood fusion (MALF) (46) algorithm, was utilized for 3D whole-brain segmentation. The MALF algorithm depends on the information of multiple atlases each of which consists of an MR brain image and a pre-defined segmentation map (46). In this study, 45 atlases were used and each had previously been segmented into a total of 289 anatomical regions including the three sub-regions of CC (gCC, bCC, and sCC). Details of the 45 atlases and the associated 289 labels can be found elsewhere (47). Before segmentation all T1-weighted images had been rigidly aligned to the MNI space (48), and thus the segmentation results were in the MNI space as well. The entire segmentation pipeline is freely available at the MriCloud platform (www.mricloud.org). Each segmentation result was visually inspected and manually corrected in case of automated segmentation error.

After obtaining the 3D whole-brain segmentation of each T1-weighted image, we extracted the binary segmentation results of gCC, sCC, and bCC and combined them to form the 3D binary segmentation of CC. We then took out the 2D mid-sagittal slice. In the MNI space, there are a total of 181 sagittal slices and thus the 2D mid-sagittal one refers to the 91st slice.

### CC Curve Generation and Template Curve Selection

To extract the boundary curve from each 2D mid-sagittal section of CC, we used a boundary following algorithm (49) to identify the boundary pixels, starting with the point at the lower-left corner of CC and then arranging the boundary pixels clockwise to form a curve representing the 2D mid-sagittal CC shape which was analyzed later.

In LDDMM-curve based statistical shape analysis, a template curve is mapped to all target curves (curves from all participants). As such, the first thing is to select a template curve. In this study, we selected the template CC curve to be the one whose area was closest to the mean area averaged across all CC curves. This template selection method had been widely adopted and validated in LDDMM-based statistical shape analyses in previous works (35, 50, 51).

### LDDMM-Curve Registration and Deformation Marker

Once we obtained the template curve, each target curve was firstly rigidly aligned to the template curve to reduce the computational cost of subsequent LDDMM-curve and improve its registration accuracy. After that, we employed LDDMM-curve to get a diffeomorphic transformation mapping the template curve to the rigidly aligned target curve. Methodologies details regarding the LDDMM-curve algorithm can be found in its original publication (37).

After obtaining the diffeomorphic transformation from LDDMM-curve, at each point of the template curve we computed the Jacobian matrix *D* and defined a “deformation marker” *J* = det(*D*) (the determinant of the Jacobian matrix *D*). The value of this deformation marker *J* corresponds to the localized shape variation of each point on the template curve. Specifically, the value of *J* larger than 1 represents outward-deformation of the target curve relative to the template curve at a local point, otherwise inward-deformation. This deformation marker was then used in our statistical comparisons. Previous studies had already widely adopted this deformation marker in quantifying localized shape morphometry (34, 52, 53).

### Statistical Analysis

For each target curve *s*, *J*_*k*_(*s*) represents the deformation marker at point *k* of the template curve. Statistical analyses were conducted using the following linear model

$$J_{k}(s)\;=\;\beta_{k,0}\;+\;\beta_{k,1}\gamma (s)\;+\;\sum_{cov}^{}\alpha_{cov}X_{cov}(s)\;+\;\varepsilon_{k}(s)$$

In this model γ(*s*) is a binary group variable: γ(*s*) equals to 1 when the target curve *s* belongs to the FES group and γ(*s*) equals to 0 when the target curve *s* belongs to the HC group. *X*_*cov*_(*s*) denotes the covariate information and we covaried for age, gender, and total intracranial volume (TIV) in this study. ε_*k*_(*s*) represents a Gaussian noise variable. For all points *k* on the template curve, we tested the null hypothesis that β_*k*, 1_ = 0. We used Fisher's method of randomization and permutation tests to quantify the statistical significance (*p*-value) of the difference between two groups under comparison (FES vs. HC within the entire group, the male group, as well as the female group) and the *p*-values were corrected for multiple comparisons by controlling the family-wise error rate (FWER) at a level of *P* ≤ 0.05. In the process of permutation tests, we generated 10,000 uniformly distributed random permutations by employing Monte Carlo simulations. We used −β_*k*, 1_ to denote the degree of a group difference, so that a positive value represents inward-deformation in the FES group relative to the HC group whereas a negative value represents expansion. We employed the same linear model and the same statistical analysis method to compare the CC area, gCC area, bCC area, and sCC area between HC and FES within the entire group, the male group, and the female group.

To investigate the interaction effect between gender and the FES status on CC's morphology (including both global area and localized shape), we first employed the above linear regression model considering also interaction effect. Specifically, the gender and group variables were included as two main factors and the age and TIV were included as two covariates. Interaction of the two main factors (gender and group) was also included in the model. Gender-specific *post-hoc* group comparison analyses would then be performed if that the interaction term was statistically significant. When analyzing each single-gender group, we only co-varied for age and TIV.

**Figure 1:**
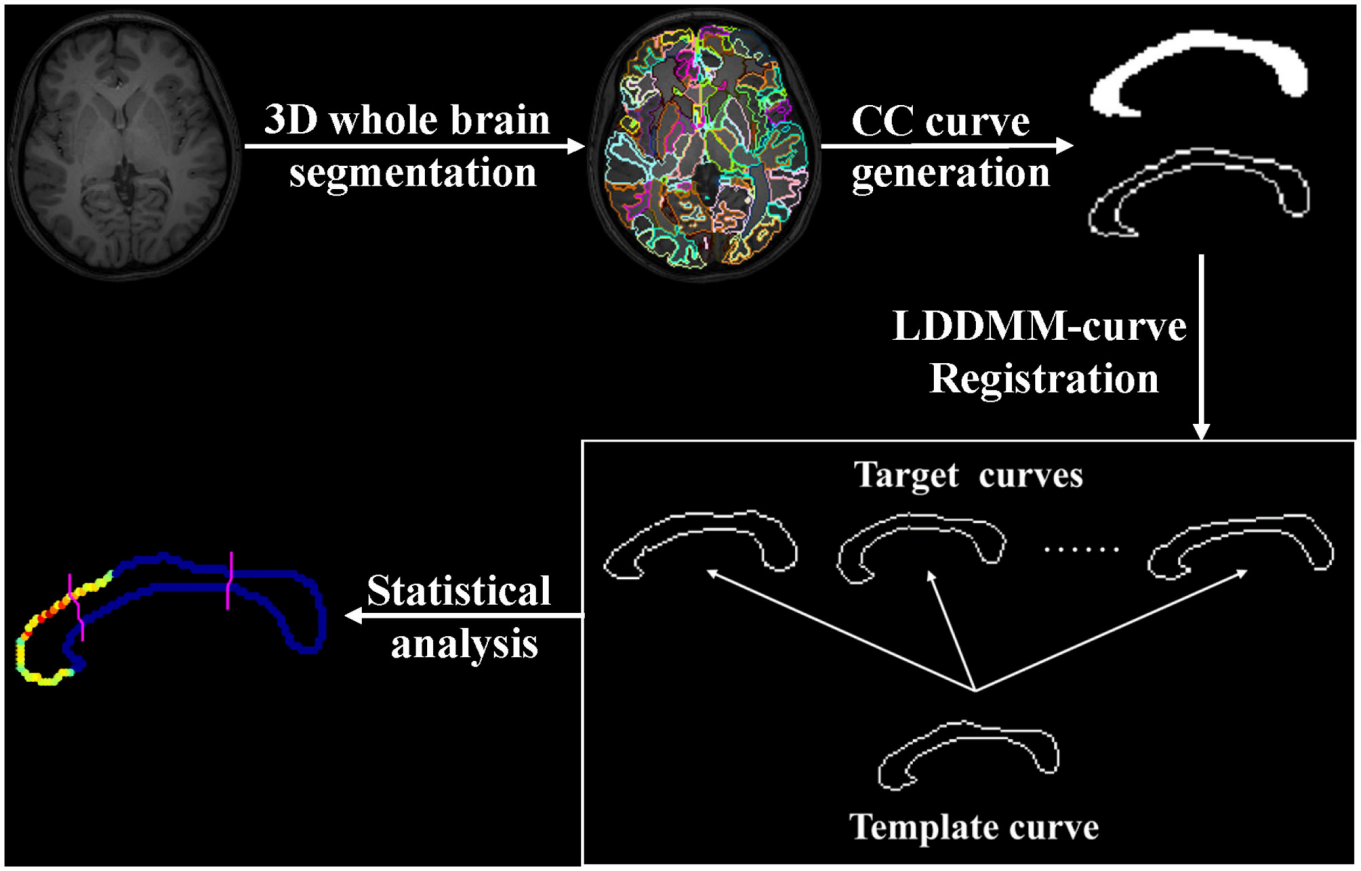
Analysis flow of the entire pipeline.

Cohen's *d* (54) was used to measure the effect size and to quantify the extent of a group difference in both global area analysis and localized shape analysis. There is a general explanation for the numerical value of Cohen's *d*: when d is around or smaller than 0.2, it suggests a small group difference; when *d* is around 0.5, it suggests a medium group difference; when *d* is larger than 0.8, it suggests a large group difference. The analysis flow of the entire pipeline is shown in Figure 1.

## Results

### Global Area Analysis

Significant gender-vs.-group interaction effect was detected in analyzing the entire CC area (*P* = 0.0106), the gCC area (*P* = 0.0072) and the sCC area (*P* = 0.0286). The area comparison results, between FES and HC within the entire group, the female group, and the male group, for CC and its three sub-regions (gCC, bCC, sCC) are listed in Table 2. Clearly, there was significant group difference between FES and HC in the entire CC area (*P* = 0.0266, *d* = 0.2941) and that difference was exclusively concentrated on the gCC sub-region (*P* = 0.0005, *d* = 0.4962). No significant FES-vs.-HC group differences were detected in the areas of the other two sub-regions, namely bCC and sCC. Looking into the gender effects, we found that the FES-related gCC area reduction was specific to female patients but not male patients. Specifically, in the female group, there was significant area reduction in FES relative to HC in the gCC sub-region (*P* = 0.0001, *d* = 0.876) but not the other two ones. The FES-vs.-HC group difference in the entire CC area within the female group approached being significant (*P* = 0.078, *d* = 0.4409). Within the male group, there was no significant FES-vs.-HC group difference in either the entire CC area or the area of each of the three sub-regions. This finding may suggest that female is more vulnerable to the FES neuropathology than male. From that table, we also observed that within either FES or HC females had larger areas than males in the entire CC and each of its three sub-regions.

**Table 2:** Area analysis results obtained from comparing FES and HC within the entire group (All), the female group (Female), and the male group (Male).

		**HC Group (mm^**2**^)**	**FES Group (mm^**2**^)**	***p*-value**	**Cohen's *d***	**Group difference (mm^**2**^)**
All	CC Area	703.88 ± 84.97	678.93 ± 84.62	0.0266	0.2941	−26.1342
	gCC Area	194.64 ± 27.79	180.76 ± 28.18	0.0005	0.4962	−14.0706
	bCC Area	291.23 ± 41.17	287.88 ± 39.25	0.5355	0.0830	−3.4701
	sCC Area	218.01 ± 37.33	210.29 ± 33.92	0.0774	0.2156	−8.5935
Female	CC Area	730.81 ± 79.52	694.68 ± 84.14	0.0780	0.4409	−29.4792
	gCC Area	203.51 ± 23.89	181.20 ± 26.86	0.0001	0.8760	−20.3965
	bCC Area	298.47 ± 44.72	292.98 ± 42.33	0.7260	0.1262	−3.2936
	sCC Area	228.83 ± 37.47	220.50 ± 31.99	0.4135	0.2397	−5.7890
Male	CC Area	682.42 ± 83.67	660.19 ± 82.27	0.1665	0.2676	−22.1695
	gCC Area	187.58 ± 28.83	180.24 ± 29.99	0.2085	0.2503	−7.4186
	bCC Area	285.46 ± 37.50	281.81 ± 34.76	0.6155	0.1002	−3.6524
	sCC Area	209.39 ± 35.20	198.14 ± 32.45	0.1060	0.3299	−11.0985

*A negative value indicates an area reduction in the disease group relative to the control group*.

### Localized Shape Analysis

**Figure 2:**
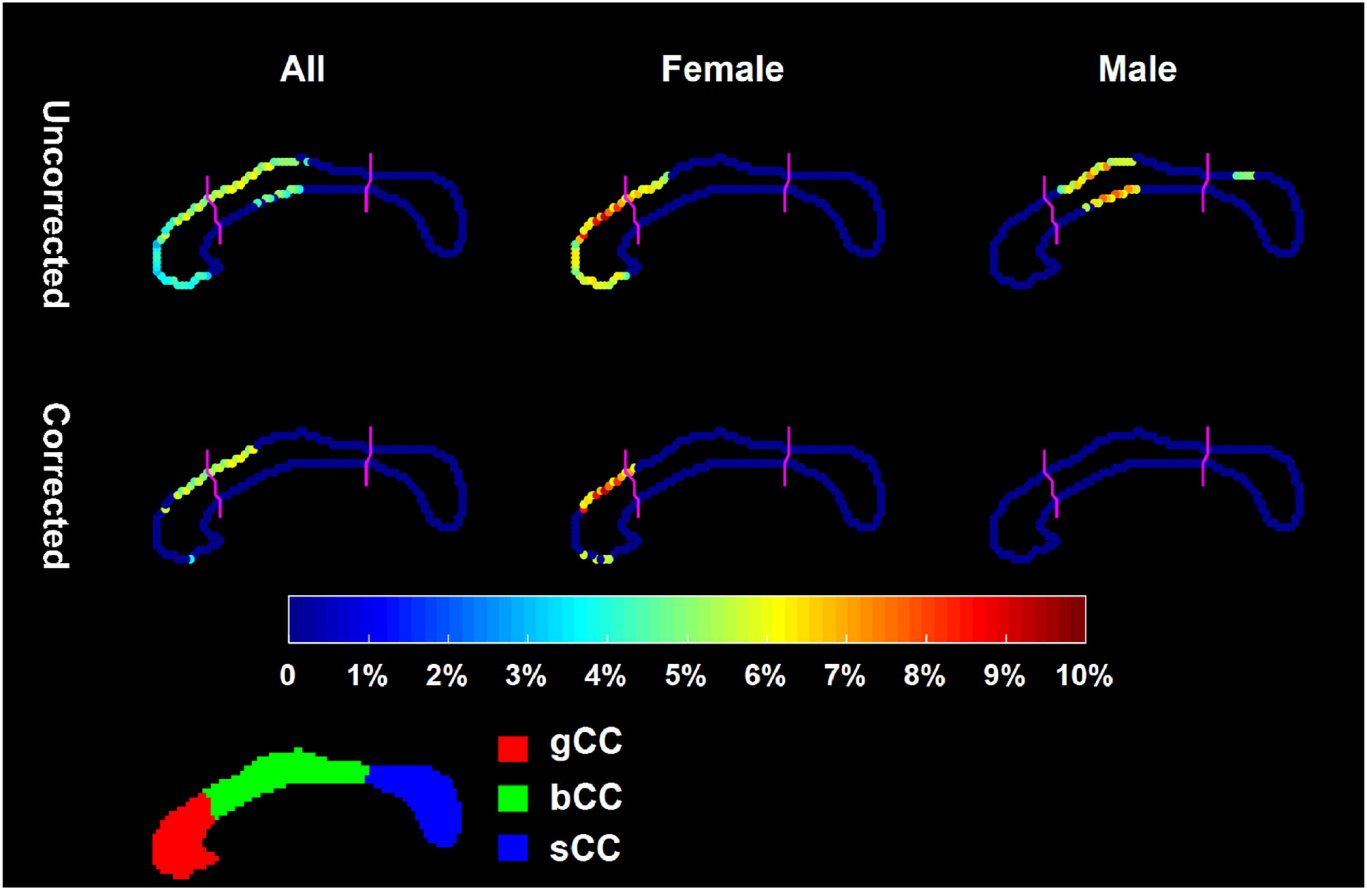
Shape analysis results of the 2D mid-sagittal CC curve within the entire group (the leftmost panel), the female group (the middle panel), and the male group (the rightmost panel). The first row demonstrates uncorrected results and the second row demonstrates the FWER corrected results. The color bar denotes the degree of inward-deformation in the patient group relative to the control group. The CC sub-region definitions are illustrated at the bottom panel.

**Figure 3:**
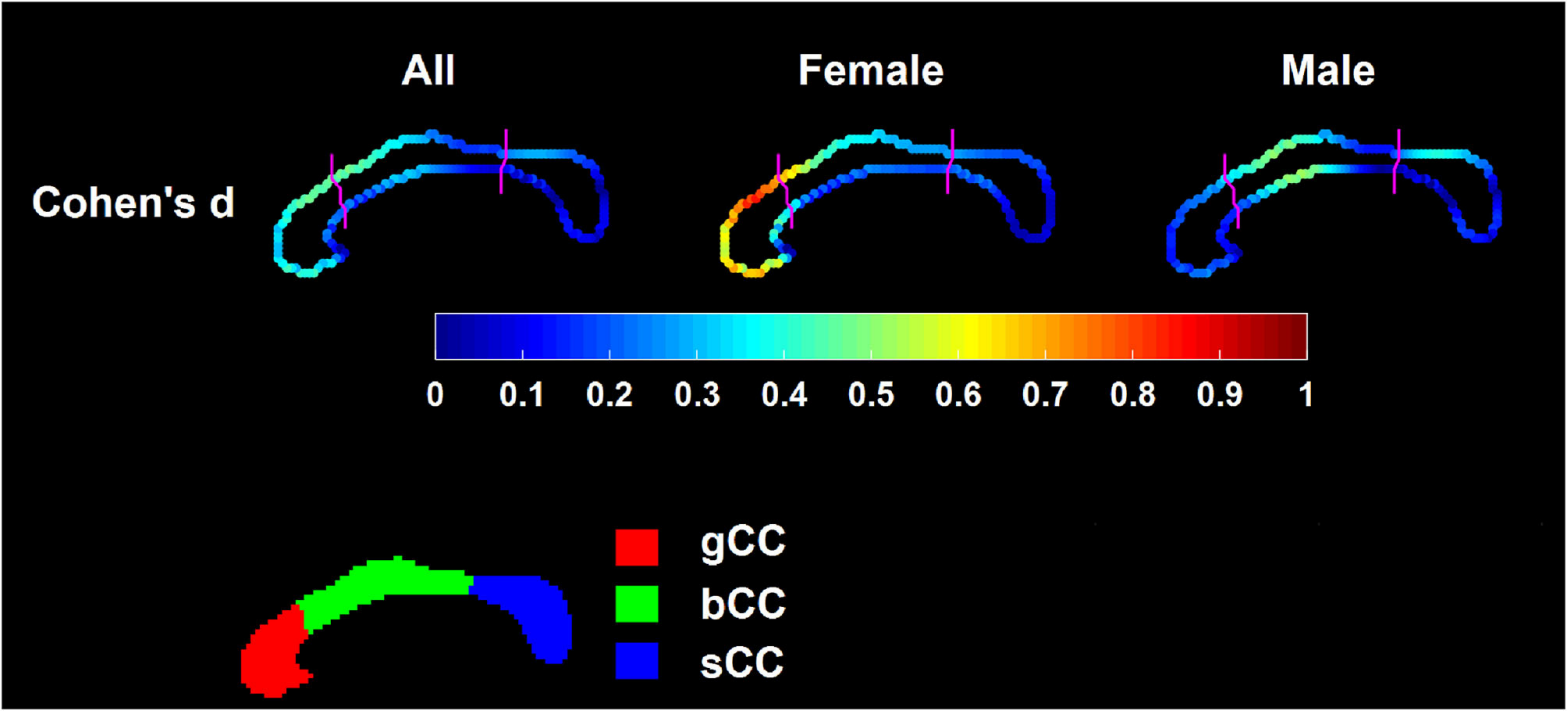
Cohen's *d* computed from point-wise deformation marker on the 2D mid-sagittal CC curve within the entire group (the leftmost panel), the female group (the middle panel), and the male group (the rightmost panel).

In our shape analysis experiments, we also found significant gender-vs.-group interaction effect (*P* = 0.0345). Being consistent with area, the localized shape of CC differed significantly between HC and FES (*P* = 0.0123). This single *p*-value reflects the significance of the overall shape difference between the two groups under comparison (33). The localized shape comparison results of CC, characterizing the point-wise group differences between HC and FES, are visualized in Figure 2 (the leftmost panel), including both uncorrected and corrected results. Figure 3 displays the corresponding Cohen's *d* results computed on the point-wise deformation markers. In Figure 2, the points highlighted denote the ones whose localized shape characteristics differed significantly between the two groups under comparison, and color of those highlighted points represents the degree of inward-deformation in the disease group relative to the control group. As revealed by that figure, after multi-comparison correction, significant FES-related region-specific inward-deformation occurred mainly at the superior portion of gCC and the antero-superior portion of bCC. Also keeping in line with area findings, there was gender effect in the localized CC shape phenotype in FES (as displayed in Figures 2, 3). Specifically, the statistically significant FES-related CC shape abnormalities were exclusive to female patients (*P* = 0.0028, *d* = 0.736 ± 0.0533) but not male ones. The region-specific CC inward-deformations in female FES were solely concentrated on the superior portion of gCC. After correction, there was no significant CC shape difference between FES and HC within the male group, being consistent with the area observation. After multi-comparison correction, the average value of Cohen's *d* averaged across points exhibiting significant FES-vs.-HC group difference was *d* = 0.4515 ± 0.021 in the entire group and *d* = 0.736 ± 0.0533 in the female group.

## Discussion

In the past few decades, numerous theoretical and empirical studies have identified correlations between CC and schizophrenia. CC is a between-hemisphere relay center. Abnormal interhemispheric transmission in CC, to some extent, may explain certain schizophrenic phenomena such as paranoid delusions and auditory hallucinations (55). In addition, not only morphological studies but also electrophysiological and neuropsychological studies have demonstrated altered CC connections in schizophrenia (56). Researches on interhemispheric electroencephalogram coherence have suggested differences in the frontal-temporal connectivity and coherence between schizophrenic patients and healthy controls (57). From the neuropsychological study of Beaumont and Dimond (58), schizophrenic patients experienced defects when confronting matched stimuli exerted separately to the left and right hemispheres, which also provided evidence for interhemispheric communication impairment in schizophrenia. Recently, a large-scale coordinated study from the ENIGMA Schizophrenia diffusion tensor imaging (DTI) Working Group (ENIGMA-SZ DTI) analyzed 2,359 healthy controls and 1,963 schizophrenia patients, and reported widespread white matter abnormalities in schizophrenia patients and the most affected regions were anterior corona radiata and CC (specifically gCC and bCC) (16). Reviewing existing literature, localized shape analyses of CC in schizophrenia, especially in FES, nevertheless have been very rare.

In this study, we conducted group comparisons between HC and FES in terms of both global and local CC morphometrics. For the global measurement, we analyzed area. For the local measurement, we analyzed shape, for which a deformation based statistical shape analysis pipeline was utilized. The statistical shape analysis pipeline employed in this work is well-established and has been validated in many previous studies (33, 36, 38). As such, the methodological novelty of this work is relatively limited. And we focus more on the scientific findings. In the analysis, CC was divided into gCC, bCC, and sCC so as to identify the key sub-regions of CC targeted by the neuropathology of FES. We also examined the gender difference in terms of FES-associated morphological abnormalities of CC by comparing HC and FES within the female group and within the male group.

The advantage of the shape analysis pipeline employed in this work is that it is fully automatic whereas existing CC analysis approaches mainly employed hand-engineered feature extraction methods. In addition, our point-wise shape deformation markers from LDDMM-curve took neighboring morphometry into consideration instead of only considering each isolated point. Previous MRI studies on CC morphometry mostly focused on its 3D global volume (29, 48, 59) or its 2D area measurement (19, 20, 25) rather than its localized shape characteristics. As far as we know, this is the first study to have investigated the influence of the neuropathology of FES exerted to the mid-sagittal CC shape using curve-based shape deformation.

Compared to other MRI-based CC analysis in FES, the sample size of our study is relatively large. More specifically, a total of 198 participants (92 FES patients and 106 healthy controls) were included in our study. Keshavan et al. (20), for instance, has only included a total of 74 participants, Takahashi et al. (31) has included a total of 92 participants and Walterfang et al. (32) has included 146 participants.

According to our global area analysis results, there was significant HC-vs.-FES group difference in the area of the entire CC and that different was specific to the gCC sub-region. This result was however only partially consistent with some previous studies (19, 20) wherein the CC area reductions were not specific to gCC but also some other sub-regions. Such inconsistency may have been due to two potential reasons: (1) The sample sizes are different from study to study. A relatively larger sample size in our current study identifies the robustness of our reported findings. (2) The sub-region definitions may be different.

In our localize shape analysis, significant FES-related region-specific shape inward-deformations were observed at the superior portion of gCC and the antero-superior portion of bCC. These shape results, to some extent, were partially consistent with those reported by several previous studies (30, 32), although they focused on regional thickness but not deformation. To investigate the relationship between our deformation marker and regional thickness, we calculated the Pearson Correlation Coefficient (PCC) between the average thickness (7.343 ± 0.732) and the average deformation marker (1.007 ± 0.088) of the entire 2D CC, and found a significant linear correlation (PCC = 0.8453, *P* < 0.0000001). This indicates that the two measurements are tightly linked, but they are not equivalent. In 2D cases, the deformation marker measures the relative area change over an infinitesimal region but not regional thickness. When averaged over a region of interest, the value of the deformation marker should be highly correlated to the area of that region. To verify this statement, we also calculated the PCC between the area (692.3 ± 85.7) and the average deformation marker (1.007 ± 0.088) of the entire 2D CC curve, with a linear correlation of 0.9869 (*P* < 0.0000001) having also been achieved. Apparently, those two measurements are highly correlated, and the correlation is much stronger than that between the deformation marker and thickness (0.9869 vs. 0.8453). Furthermore, we compared the area of each CC curve with the result of multiplying the area of the template CC curve with the average target-specific deformation marker (one for each CC curve), with 692.3 ± 85.7 and 704.7 ± 145.6 having been obtained, respectively, for the former and the latter. No significant group difference was found between those two sets of measures (*P* = 0.3044 from Student's *t*-test).

It is noteworthy that we found remarkable gender effects in our findings. Specifically, we observed that in both HC and FES females had larger areas than males in the entire CC and each of its three sub-regions, which agreed with previous findings (19, 30, 42) and that female patients had larger CC area reductions than male patients when compared to their healthy control counterparts, especially in the gCC area. In our morphometric analyses, only the female group exhibited statistically-significant global and local inward-deformations in gCC, but not the male group. Those gender differences may indicate that the CC morphology, especially the gCC morphology, is more vulnerable to the FES neuropathology in female than in male. Therefore, the global area and localized shape characteristics of gCC may serve as novel and efficient biomarkers for screening female FES patients.

gCC is the most curved part of the anterior CC, as illustrated in the two figures of this work. The forceps minor is a tract projecting fibers from the genu. It connects the medial and lateral surfaces of the frontal lobe and is functionally related to the frontal lobe (60). Therefore, gCC is considered to be involved in emotional regulation and its ventral side is closely related to attention and mood processing which may, to some extent, explain the FES-associated gCC abnormality observed in our study.

The gender difference in schizophrenia may be particularly complex, which may be caused by numerous factors, such as the social environment, genetic and hormonal differences, differences in brain maturity and morphology, as well as age and gender-specific behavior patterns. Previously, Häfner (61) discovered that the only significant difference between men and women was the age at illness onset and at all the following milestones of the early illness course, and this difference seems to be due to the protective effect of estrogen. When estrogen secretion starts waning in preclimacterium, women begin to show a second peak of onsets, exhibiting more severe forms of first-episode symptoms and a more severe course of the illness than men. From one perspective, this may, to some extent, explain why morphometric abnormalities in gCC were only observed in female FES but not male FES in our study.

How the FES neuropathology affects the morphology of CC is still an on-going research topic and more investigations from different disciplines are needed to completely unveil the underlying mechanism. The goal of this work is not to fully unveil the secrets of FES from the CC viewpoint. Instead, we believe that the findings reported in this work can provide some new insights into the FES field. More future works and research efforts are needed from multiple disciplines.

There are still limitations in this current study. For example, other subtypes of schizophrenia, such as chronic schizophrenia and established schizophrenia, could have been included in our comparative analyses to deliver a more comprehensive understanding of the pathology of schizophrenia. In addition, the method of choosing the mid-sagittal slice employed in this study cannot well guarantee that the slice identified is truly mid-sagittal (62). A potential solution is to design a key frame identification method (63) borrowing insights from the computer vision field, which we are currently working on. Furthermore, the age range of the population studied in this work is relatively wide, namely 12–43 years. Apparently, the brain of a 12-year-old person can be quite different from that of a 43-year-old person, which may make our sample within the same category (HC or FES) inhomogeneous. A good solution is to separate data by a few age cohorts so that they can be analyzed separately. However, our sample size is kind of limited (92 FES and 106 HC), which does not allow such division. We have already started collecting MRI data from participants of different age cohorts, and would consider age-specific analyses as one of our future research plans. Another limitation of this work is that we focused solely on the morphometrics of 2D CC but not 3D CC which nevertheless may be also a biomarker of interest in FES. We will consider analyzing 3D CC in FES as one of our future works, both globally (3D volume) and locally [statistical shape analysis for surfaces (64)]. Anticipating future research directions, it is also worth investigation the symptomatology association between CC morphology (both globally and locally) and FES pathology in males vs. females.

## Data Availability Statement

The raw data supporting the conclusions of this article will be made available by the authors, without undue reservation.

## Ethics Statement

The studies involving human participants were reviewed and approved by The First Affiliated Hospital of Shenzhen University Ethics Committee. Written informed consent to participate in this study was provided by the participants' legal guardian/next of kin.

## Author Contributions

XT designed the entire study and the analysis pipeline. WH and MC performed all experiments and associated statistical analyses. WH wrote the first draft of the manuscript. GL collected MRI data. All authors contributed and approved the final manuscript.

## Conflict of Interest

The authors declare that the research was conducted in the absence of any commercial or financial relationships that could be construed as a potential conflict of interest.
